# Supplementary material for: A comprehensive interpretable machine learning framework for mild cognitive impairment and Alzheimer’s disease diagnosis
Source: Sci Rep. 2025 Mar 11;15:8410. doi: 10.1038/s41598-025-92577-6 (PMC11897299; doi:10.1038/s41598-025-92577-6)
Supplement: Supplementary file 1 — Supplementary Information. [file 41598_2025_92577_MOESM1_ESM.pdf]

Supplementary Material

Dataset

Age	CN	MCI	AD
**60-69 (F/M)**	96 (60.42%/39.58%)	236 (48.31%/51.69%)	55 (40.00%/60.00%)
**70-79 (F/M)**	287 (50.18%/49.82%)	366 (34.15%/65.85%)	151(46.36%/53.64%)
**80-86 (F/M)**	66 (33.33%/66.67%)	138 (41.31%/58.69%)	68 (32.35%/67.65%)
**Total (F/M)**	449 (49.89%/50.11%)	740 (40.00%/60.00%)	274 (41.61%/58.39%)

Table S1 - Distribution of classes per age range and the respective percentage of male and female participants in each class

Classifiers' hyperparameters
Hyperparameters' tuning was performed using the grid search technique within the applied nested cross fold validation scheme towards the identification of the optimal hyperparameters' combination for each of the six ensemble classifiers. The hyperparameters' combinations which achieved the highest performance in terms of the balanced accuracy and F1-scores are presented in Table S2.

	Optimal Hyperparameters' Combinations
**RF**	(estimators = 600, maximum depth = 100, minimum samples leaf = 4, minimum samples split = 5)
**LR**	(penalty = L2, C = 0.01, solver = 'lbfgs,' maximum iterations = 100)
**MLP**	(hidden layer sizes = [50, 100 and 50], activation function = ReLu, solver = SGD, alpha = 0.05, learning rate = constant)
**SVM**	(C = 1, gamma = 0.00, kernel = 'rbf')
**GB**	(estimators = 500, maximum depth = 3, learning rate = 0.01)
**XGB**	(learning rate = 0.05, maximum tree depth = 3, minimum child weight = 1, gamma = 0.3, column sample by tree = 0.5)

Table S2 - Optimal hyperparameter combinations for the six different classifiers (Random Forest, Logistic Regression, Support Vector Machines, Multilayer Perceptron, Gradient Boosting, XGBoost) based on the mean balanced accuracy metric obtained by the hyperparameter tuning process of the nested cross fold validation.

Random Forest Gini Importance
As shown in Figure S1, during the binary classification between CN and MCI classes, the five most important features included the following ROI volumes: the left and right hippocampus, the left entorhinal area, the left middle temporal gyrus, and the right amygdala. A great majority of the SNPs appeared at the bottom of the feature ranking and were shown to play a minimal role in the distinction between the two classes. However the rs429358 SNP, which is located in the Apolipoprotein E gene (ApoE) [1], was ranked within the first 15 places of feature importance. When distinguishing between the MCI and AD classes, the most prevailing features were the right inferior temporal gyrus, the left lateral ventricle, the left hippocampus, the left inferior temporal gyrus, and the right middle temporal gyrus, while all the SNP features were ranked as least important. In the distinction between the CN and the AD classes, the higher ranked ROIs were the

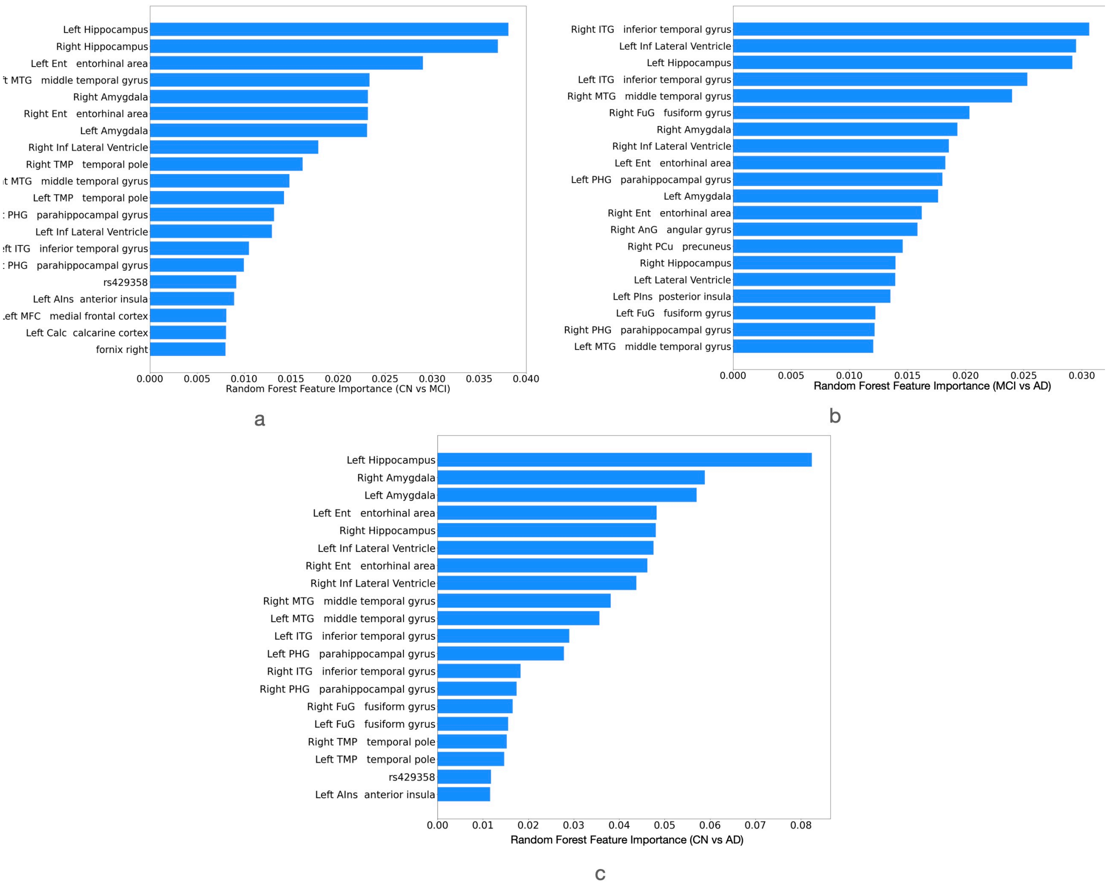

Figure S1 - Random Forest model Feature Importance ranking for the classification of the a) Control (CN) and Mild Cognitive Impairment (MCI) classes b) MCI and AD classes and c) CN and AD classes

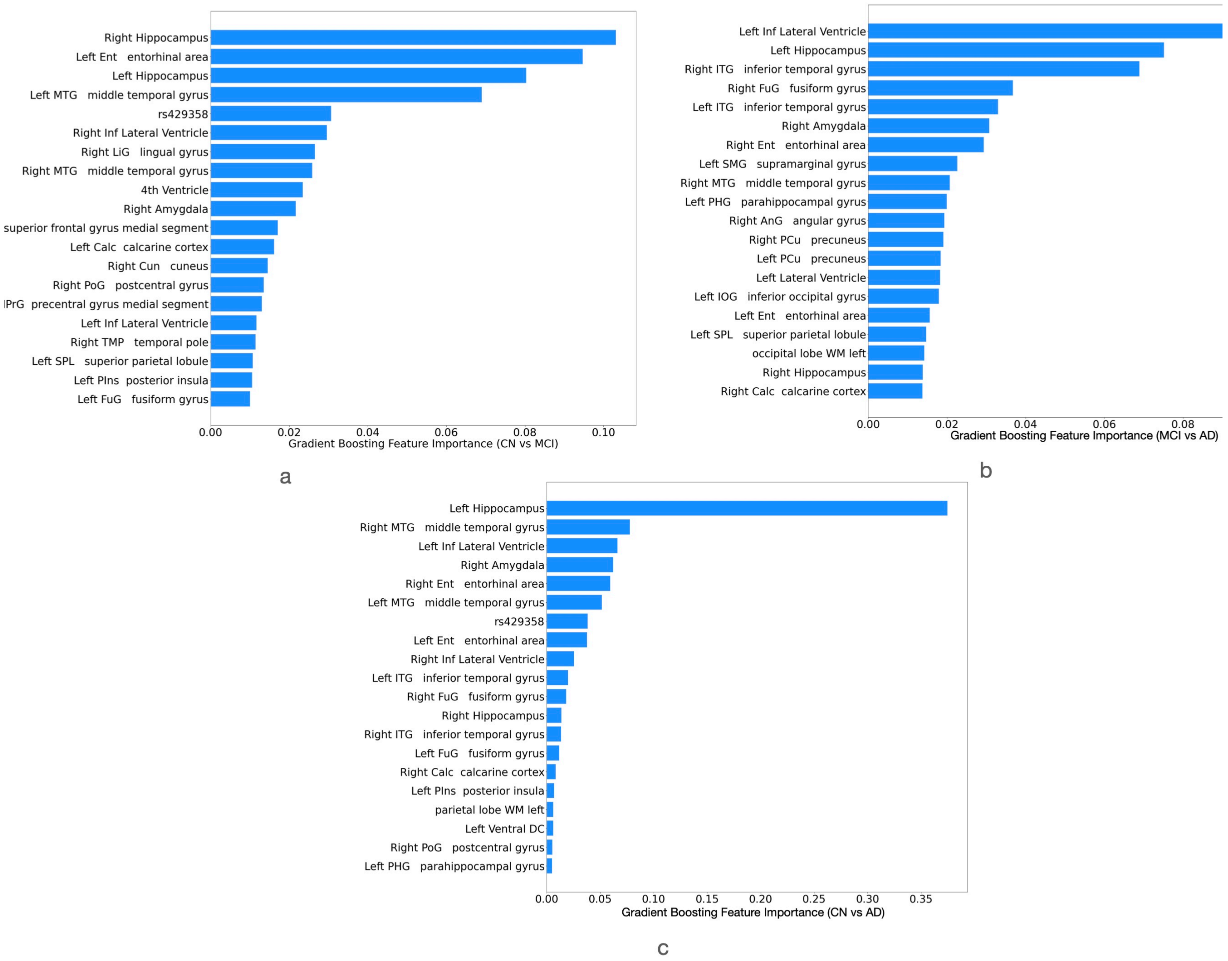

Figure S2 - Gradient Boosting model Feature Importance ranking for the classification of the a) Control (CN) and Mild Cognitive Impairment (MCI) classes b) MCI and AD classes and c) CN and AD classes

left hippocampus, the right and left amygdala, the left entorhinal area, and the right hippocampus. For this pair of classes, the rs429358 SNP was also among the top 20 highly ranked features, contrary to the rest SNP features, which were ordered at the bottom of the feature importance ranking.

Gradient Boosting Gini Importance

Since the Gradient Boosting model uses decision trees as base learners, a feature importance ranking can be obtained similarly to the Random Forest classifier. The ranking was derived by training a Gradient Boosting classifier with the optimal hyperparameters for all three pairs of classes. As depicted in Figure S2, the primary features for the distinction between the CN and MCI classes included the right hippocampus, the left entorhinal area, the left hippocampus, the left middle temporal gyrus, and the right lateral ventricle, among other ROI features, as well as the rs429358 SNP, which belongs to the ApoE gene and comprises the only SNP feature present in the ranking. Similar results were observed about the MCI versus AD and CN versus AD subproblems. The most important features for the MCI and AD pair consisted of the left lateral ventricle, the left hippocampus, the right and left inferior temporal gyrus, and the right fusiform gyrus. For the CN versus AD classes, the most important features were the left hippocampus, the right middle temporal gyrus, the left lateral ventricle, the right amygdala, and the rs429358 of the ApoE gene, among others.

XGBoost Gini Importance

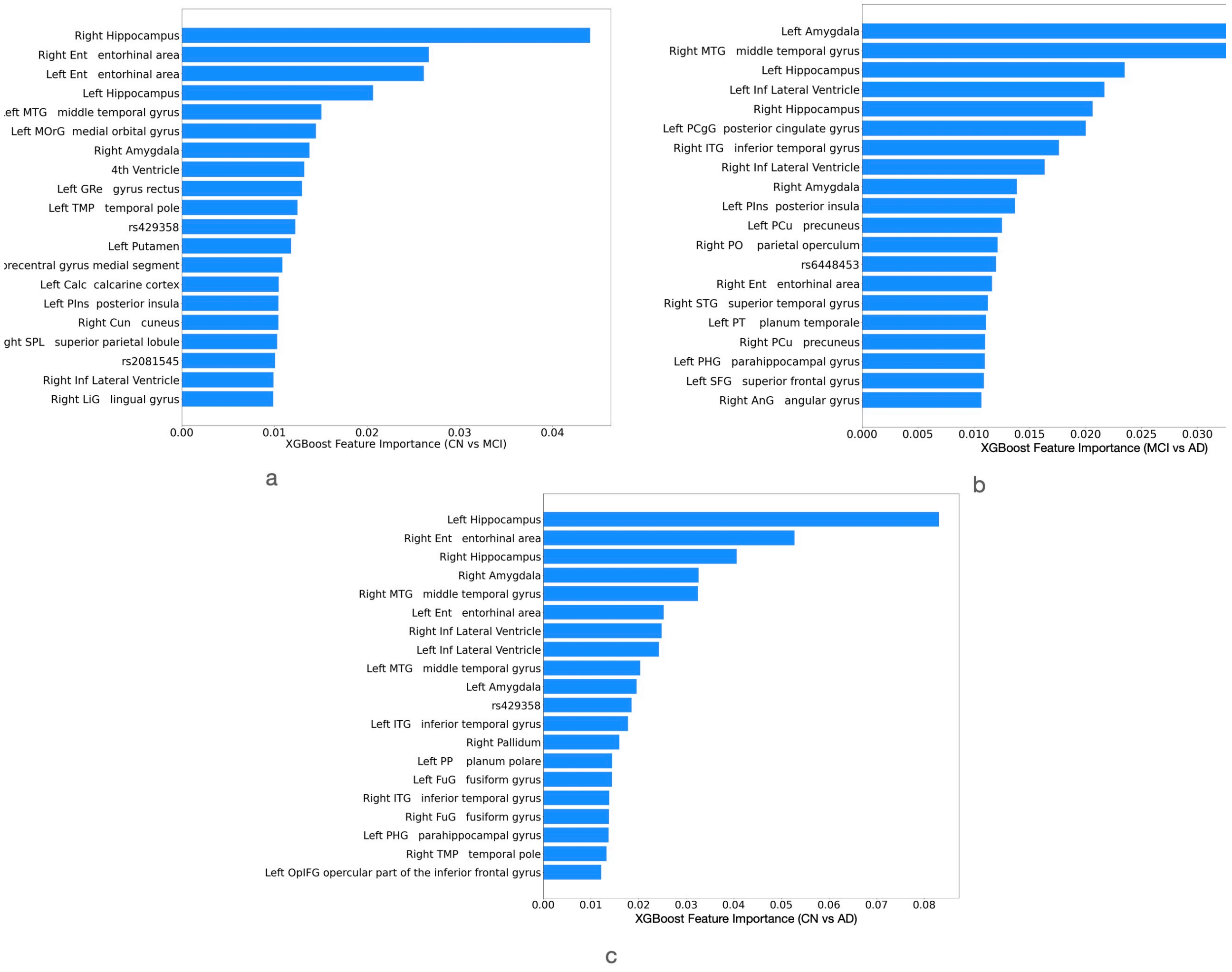

Figure S3 - XGBoost model Feature Importance ranking for the classification of the a) Control (CN) and Mild Cognitive Impairment (MCI) classes b) MCI and AD classes and c) CN and AD classes

A feature importance ranking for the XGBoost model was obtained in a manner similar to both Gradient Boosting and Random Forests. An XGBoost model with the optimal hyperparameters was trained and applied separately to each one of the three binary subproblems. According to the produced feature ranking presented in Figure S3, the most important features which the XGBoost used to distinguish between the CN and MCI classes were the right and left hippocampus, the right and left entorhinal area, and the left middle temporal gyrus. Contrary to the Random Forest feature importance ranking, that highlighted the rs429358 SNP among the most significant features, the rs2081545 SNP, which is associated with the MS4A6A gene [2], was included in the first positions of the XGBoost ranking. In terms of the MCI vs AD subproblem, the most important features comprised the right amygdala, the right middle temporal gyrus, the left hippocampus, the left lateral ventricle, and the right hippocampus. The only SNP ranked among the twenty most important features was rs6448453, which is associated with at the CLNK gene [2]. Additionally, in the distinction between the CN and AD classes, the most important features coincided with the previous rankings, since they consisted of the left and right hippocampus, the right entorhinal area, the right amygdala, and the right middle temporal gyrus, among other important ROIs. Interestingly, in this binary subproblem, the only SNP included in the twenty most important features, obtaining the eleventh position, was the rs429358, which is located at the ApoE gene.

SHAP Feature Importance

For obtaining feature importance rankings, the SHAP framework was also utilised. By calculating the mean absolute SHAP values of each feature, the features are ordered in decreasing order of importance. Figure S4 depicts the feature ranking provided by the KernelSHAP Explainer for the three binary subproblems when using an SVM classifier with the optimal hyperparameters. The most important features were similar to the previously highly ranked features, including the left and right hippocampus, the left parahippocampal gyrus, the left amygdala, the left entorhinal area, and other ROIs, such as the right lateral ventricle or the left middle occipital gyrus. All the features obtaining the first twenty positions of the ranking comprised solely of ROI features.

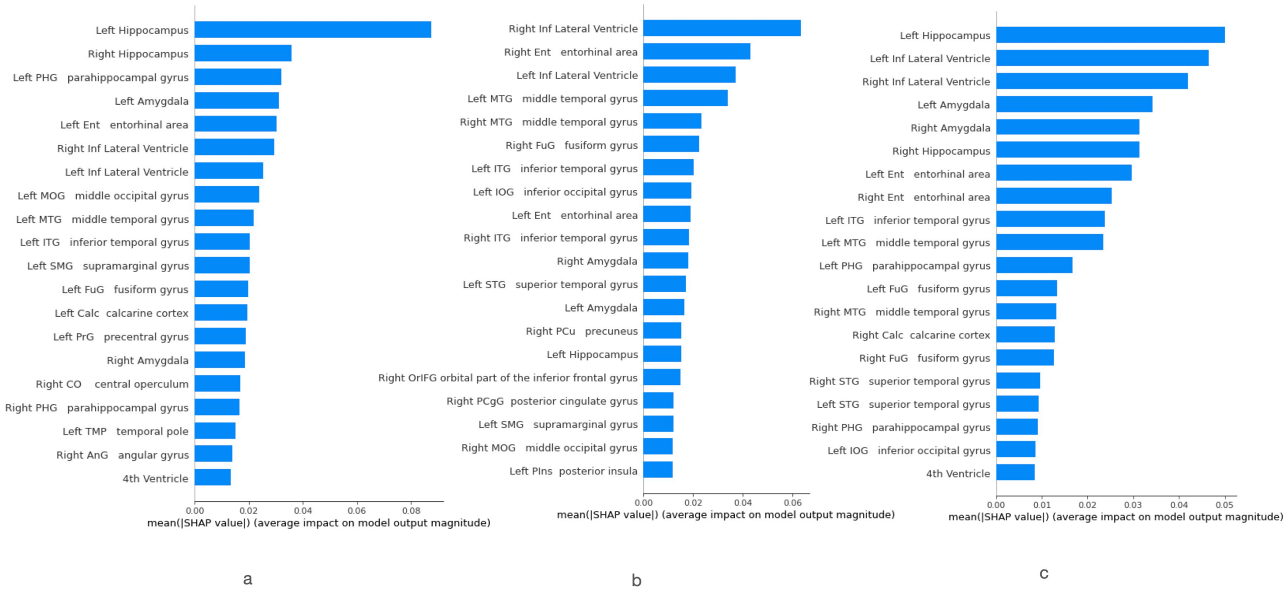

Figure S4 - Support Vector Machines model Feature Importance ranking for the classification of the a) Control (CN) and Mild Cognitive Impairment (MCI) classes b) MCI and AD classes and c) CN and AD classes, by calculating the mean absolute SHAP values of each feature with the use of KernelSHAP Explainer

SHAP Summary Plot

The Summary Plot depicted in Figure S5 was obtained by the Kernel Explainer from the classification of the three pairs of classes with a SVM Classifier. Every point represents the SHAP value of an instance, with red (blue) points indicating a high (low) feature value. Negative values

on the x-axis correspond to negative SHAP values for a specific feature, meaning this feature contributes to the instance being classified in the negative class and vice versa. The lower volume values of most of the areas of the brain appeared to contribute to brain atrophy and consequently, AD, whereas the higher volume of the left and right lateral ventricles, namely their enlargement, was shown to have a similar effect.

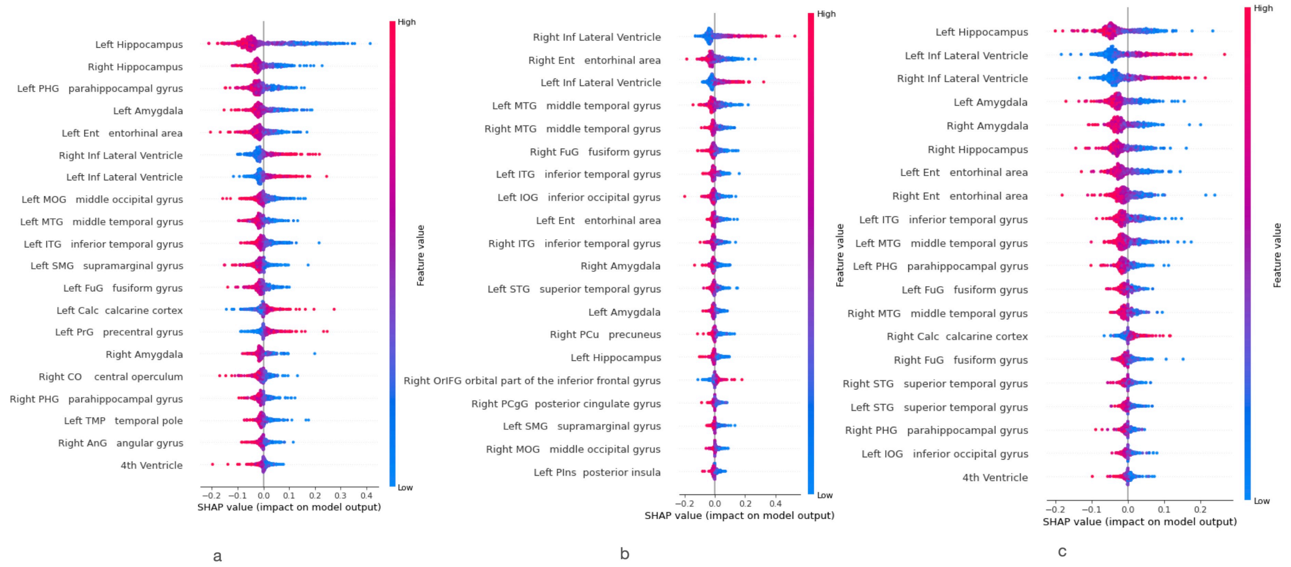

Figure S5 - SHAP method Summary plot for the classification of the a) Control (CN) and Mild Cognitive Impairment (MCI) classes b) MCI and Alzheimer's Disease (AD) classes and c) CN and AD classes with a Support Vector Classifier. Every point represents the SHAP value of an instance and when a point is depicted as red, it represents a high feature value, whereas a blue point indicates a low value. When a data point is located on the left of the y-axis, it represents an instance with a negative SHAP value for the specific feature, meaning this feature contributes to the instance being classified in the negative class (CN or MCI accordingly) and vice versa. Y-axis also represents a feature importance ranking.

Local Interpretable Model-agnostic Explanations (LIME)

The LIME algorithm was applied separately on each binary subproblem after the classification with the use of various classifiers.

CN versus MCI

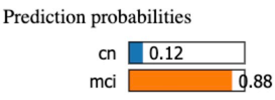

Feature	Value
Left Hippocampus	-3.35
Left Ent entorhinal area	-4.20
Left PHG parahippocampal gyrus	-4.12
Right Hippocampus	-2.53
Left Amygdala	-3.79
Left TMP temporal pole	-2.40
Left Inf Lateral Ventricle	2.56
Right Amygdala	-2.86
Right Ent entorhinal area	-1.63
Right Inf Lateral Ventricle	2.23

Figure S6 - LIME model output for Random Forest classification of the Control (CN) and the Mild Cognitive Impairment (MCI) classes. True Positive instance, where a MCI patient is correctly classified. Left: the predicted probability of the specific instance for the two classes, Middle: features that push the prediction to the MCI class (orange) and to the CN class (blue) respectively, Right: a feature importance ranking and the feature values of the presented features. Orange highlighted features push the prediction to the MCI class and features highlighted in blue push the prediction to the CN class.

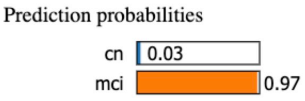

Feature	Value
Left Hippocampus	-3.35
Right Hippocampus	-2.53
Right Inf Lateral Ventricle	2.23
Left Inf Lateral Ventricle	2.56
Left Amygdala	-3.79
Left Ent entorhinal area	-4.20
Left PHG parahippocampal gyrus	-4.12
Left ITG inferior temporal gyrus	-3.04
Left MOG middle occipital gyrus	0.64
Left FuG fusiform gyrus	-1.35

Figure S7 - LIME model output for Support Vector Machines classification of the Control (CN) and the Mild Cognitive Impairment (MCI) classes. True Positive instance where a MCI patient is correctly classified. Left: the predicted probability of the specific instance for the two classes, Middle: features that push the prediction to the MCI class (orange) and to the CN class (blue) respectively, Right: a feature importance ranking and the feature values of the presented features. Orange highlighted features push the prediction to the MCI class and features highlighted in blue push the prediction to the CN class.

For the distinction between the CN and MCI classes, local explanations were obtained for four different individuals, one True Positive, one True Negative, one False Positive and one False Negative randomly selected instance, with CNs being considered as the “negative” samples and MCI patients as the “positive” ones. Based on the obtained results for the same MCI subject using the Random Forest and SVM classifiers (Figures S6 & S7 respectively), the values of the left and right hippocampus, the left entorhinal area, the left parahippocampal gyrus, and the right and left lateral ventricle volumes, among other ROI features, appeared to increase a sample’s probability of belonging to the MCI rather than the CN class. The most prominent feature that increased a sample’s probability of belonging to the CN class was the left middle occipital gyrus. Most of the aforementioned features aligned with the generated feature rankings for this pair of classes based on the Gini importance and the SHAP method (Figures S1-S4). Between the two classifiers, slightly differentiated explanations and predictions were observed for the same patients.

MCI versus AD

As for the distinction between the MCI and AD classes, the explanations obtained by the LIME model showed that the ROI volumes that increased a sample’s probability of belonging to the AD rather than the MCI class, were, among others, the right middle temporal gyrus, the right inferior temporal gyrus, the right fusiform gyrus, the left hippocampus, and the right amygdala. These explanations were in accordance with the results obtained from feature ranking for the same pair of classes. On the other hand, the features that increased the sample’s probability of belonging to the MCI class were, among others, the right and left entorhinal areas and the right lateral ventricles. Figures S8 & S9 show the explanations obtained from the LIME model for the same AD patient, by using the Random Forest and SVM classifiers, that both produced false negative predictions, classifying the subject as a MCI patient instead of AD.

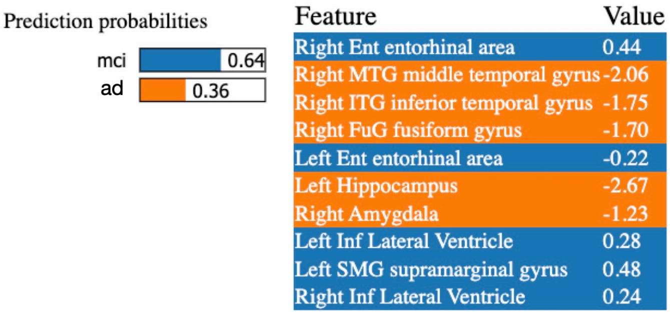

Figure S8 - LIME model output for Random Forest classification of the MCI and AD classes. False Negative instance, where an AD individual is classified in the MCI class. Left: the predicted probability of the specific instance for the two classes, Middle: features that push the prediction to the AD class (orange) and to the MCI class (blue) respectively, Right: a feature importance ranking and the feature values of the presented features. Orange highlighted features push the prediction to the AD class and features highlighted in blue push the prediction to the MCI class.

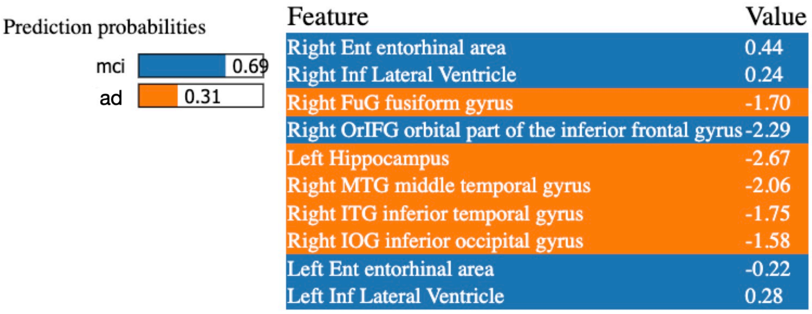

Figure S9 - LIME model output for Support Vector Machines classification of the MCI and AD classes. False Negative instance, where an AD individual is classified in the MCI class. Left: the predicted probability of the specific instance for the two classes, Middle: features that push the prediction to the AD class (orange) and to the MCI class (blue) respectively, Right: a feature importance ranking and the feature values of the presented features. Orange highlighted features push the prediction to the AD class and features highlighted in blue push the prediction to the MCI class.

CN versus AD

Regarding the classification between CN and AD subjects, the LIME model indicated that a sample’s probability of being classified as an AD patient was increased based on the volumes of the left and right hippocampus, the right lateral ventricle, and the right fusiform gyrus. The features driving the prediction towards the CN class were the right and left entorhinal areas, the left middle temporal gyrus, and the absence of the rs429358 SNP, which belongs to the ApoE gene. These explanations aligned with the feature ranking obtained by the Gini importance measure. Figures S10 and S11 present the LIME explanations provided by the two classifiers for a

health individual. While the Random Forest classifier made a correct negative prediction, the SVM classifier mistakenly classified the healthy individual as an AD patient.

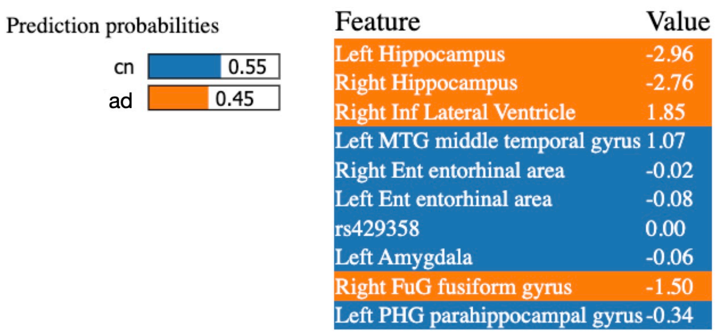

Figure S10 - LIME model output for Random Forest classification of the Control (CN) and Alzheimer's Disease (AD) classes. True Negative instance, where a healthy (CN) individual is correctly classified. Left: the predicted probability of the specific instance for the two classes, Middle: features that push the prediction to the AD class (orange) and to the CN class (blue) respectively, Right: a feature importance ranking and the feature values of the presented features. Orange highlighted features push the prediction to the AD class and features highlighted in blue push the prediction to the CN class.

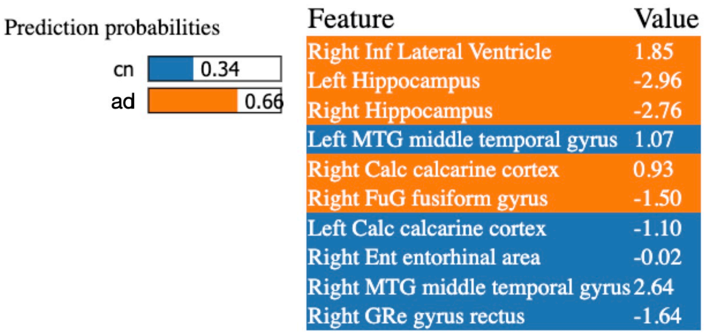

Figure S11 - LIME model output for Support Vector Machines classification of the Control (CN) and Alzheimer's Disease (AD) classes. False Positive instance, where a healthy (CN) individual is classified in the AD class. Left: the predicted probability of the specific instance for the two classes, Middle: features that push the prediction to the AD class (orange) and to the CN class (blue) respectively, Right: a feature importance ranking and the feature values of the presented features. Orange highlighted features push the prediction to the AD class and features highlighted in blue push the prediction to the CN class.

Partial Dependence Plots

The Partial Dependence Plots (PDP) framework was applied to each of the three binary subproblems both for the SVM and and the Random Forest classifier with the optimal estimated hyperparameters, towards indicating how the average prediction changes when a certain feature is changed. For every subproblem, the partial dependence plots of several important features

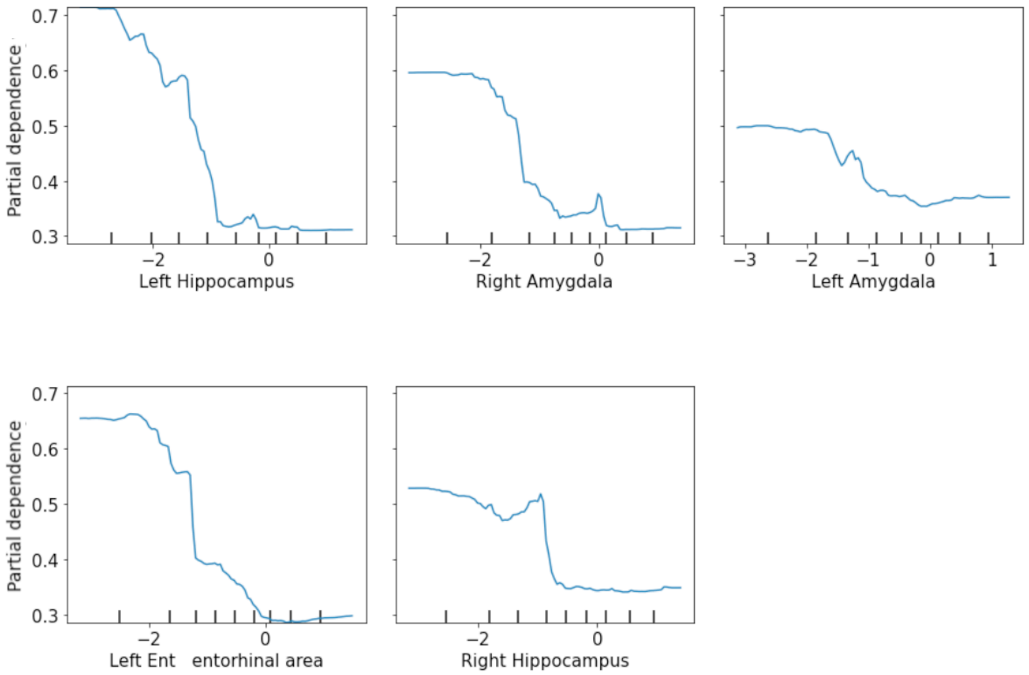

Figure S12 - Partial Dependence Plots of five important features for classification of the Control (CN) and Alzheimer's Disease (AD) classes using the Random Forest classifier. X-axis represents the values of the various features and y-axis represents the effect of this value to the probability of the AD class, meaning these plots show the effect of each specific feature to the prediction.

were derived. It was again confirmed that the smaller the volume of specific areas, such as the hippocampus, the amygdala or the entorhinal area, the greater the probability of an individual belonging to the AD class. Figure S12 depicts the partial dependence plots of certain important ROI features derived from the classification between the CN and the AD classes by the Random Forest classifier.

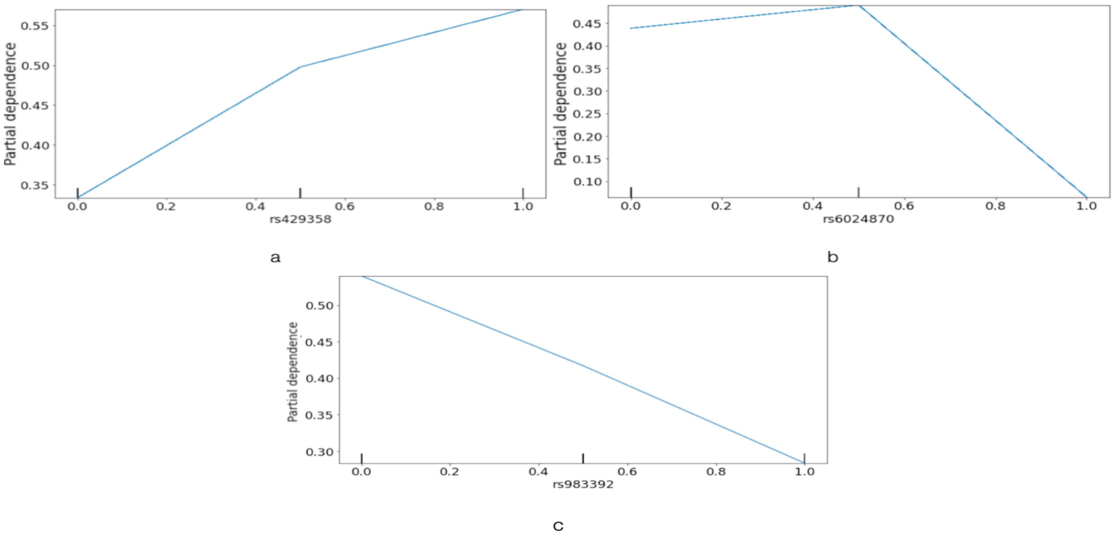

Figure S13 - Partial Dependence Plot showing the influence of a) rs429358 SNP, which belongs to the ApoE gene [1] in AD class membership from the classification of Mild Cognitive Impairment (MCI) and Alzheimer's Disease (AD) classes, b) rs6024870 SNP, which belongs to the CASS4 gene [3] in AD class membership from the classification of Mild Cognitive Impairment (MCI) and Alzheimer's Disease (AD) classes and c) rs983392 SNP, which belongs to the MS4A6A [2] gene in Mild Cognitive Impairment (MCI) class membership from the classification of Control (CN) and MCI classes. Value 0.5 in the x-axis is associated with the presence of one allele and value 1 is associated with two alleles.

The PDP were also utilised in order to study the relationship of selected SNPs with the classification output, when using the Random Forest classifier as shown in Figure S13. As already mentioned, certain SNPs were found to enhance an individual's probability of having been diagnosed with MCI or AD, whereas the presence of others seemed to lower this probability, such as the rs983392 SNP, which belongs to the MS4A6A gene [2] and was observed to lower an individual's probability of belonging to the MCI rather than the CN class when present with one or two alleles, or the rs6024870 SNP, which is associated with the CASS4 gene [3] and was observed to lower an individual's probability of belonging to the AD rather than the MCI class. The PDP of the aforementioned SNPs for the SVM classifier were not able to reliably capture the relationship of the SNPs to the prediction, as opposed to the classification with Random Forests, since the importance of the specific features was significantly lower for the former classifier as compared to their importance for the tree-based classifiers.

Permute Attack Counterfactual Explanations

For the CN and MCI pair of classes, one counterfactual example was obtained for every instance in the test set and the number of times each feature was altered in order to contribute to the class overturn, was calculated. The obtained frequency of value alterations for each feature in the generated counterfactuals, considered as an indicator of the feature's importance, is depicted in Figure S14. It can be observed that an increase in the patient's hippocampus, parahippocampal gyrus, middle temporal gyrus or amygdala and a decrease on their lateral ventricles seems to contribute to the overturn of the MCI prediction and result in the individual's classification to the CN class.

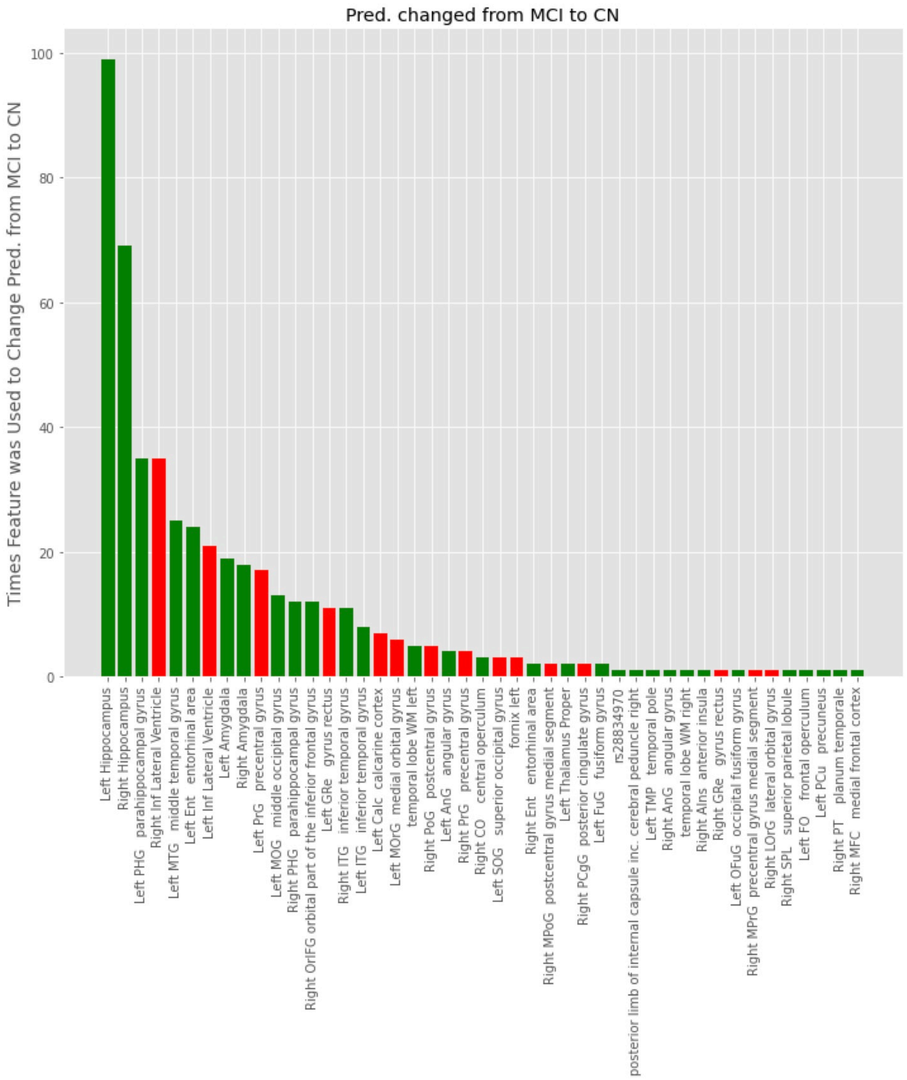

Figure S14 - Bar plot showing how many times a feature was selected to be modified in the counterfactual examples, which were created for the test set of the Mild Cognitive Impairment (MCI) and Control (CN) classes. The features are ordered in descending order of frequency and a green bar represents an overall positive feature value change (increase) and a red bar an overall negative feature value change (decrease).

References

1. Alavez-Rubio, S., Martínez-Rodríguez, N., Escobedo-De-La-Peña, J., Garrido-Acosta, O. & Juárez-Cedillo, T. Relationship Between Genetic Variants of ACAT1 and APOE with the Susceptibility to Alzheimer's disease (SADEM Study). (2020) doi:10.1007/s12035-020-02162-3.
2. Jansen, I. E. et al. Genome-wide meta-analysis identifies new loci and functional pathways influencing Alzheimer's disease risk. *Nature Genetics* **51**, 404–413 (2019).
3. Andrews, S. J., Fulton-Howard, B. & Goate, A. Protective Variants in Alzheimer's Disease. *Current Genetic Medicine Reports* **7**, 1–12 (2019).
